# Acne accounts for an almost 2.5-fold higher proportion of dermatology visits among adult females compared to adult males in the United States: A study of the national ambulatory medical care survey from 2002–2016

**DOI:** 10.1371/journal.pone.0290763

**Published:** 2023-09-21

**Authors:** Jungsoo Chang, Michael R. Nock, Jeffrey M. Cohen, Christopher G. Bunick

**Affiliations:** 1 Department of Dermatology, Yale School of Medicine, New Haven, Connecticut, United States of America; 2 Program in Translational Biomedicine, Yale School of Medicine, New Haven, Connecticut, United States of America; Cairo University - Faculty of Pharmacy, EGYPT

## Abstract

**Background:**

Acne vulgaris affects a significant number of females into adulthood. Juvenile acne and adult acne have different presentations and potentially distinctive pathogeneses. However, patterns in treatments specifically related to the adult female population have previously not been studied.

**Methods:**

Retrospective database analysis of healthcare utilization and medications prescribed for acne using the National Ambulatory Medical Care Survey (NAMCS) data from 2002–2016 was performed.

**Results:**

After age 20, acne accounted for an almost 2.5-fold higher proportion of dermatology visits among females compared to males (10.1% vs. 4.1%, P < 0.001). Tetracycline-class antibiotics were the most prescribed therapy within all age groups of females between 2002–2016. However, there was also a substantial rise in prescriptions of spironolactone beginning in 2012.

**Conclusion:**

A significantly greater proportion of dermatology visits by adult females are for acne in comparison to adult males. Tetracycline-class antibiotics remain the most prescribed therapy in adult age groups despite a potentially different pathogenesis of adult acne. Therefore, there is a need for further studies comparing the effectiveness of therapies specifically for adult female acne.

## Introduction

Acne vulgaris affects a significant number of adult females, and a majority of females in their twenties self-report having acne [1]. The disfiguring effects of acne can have a profound impact on all aspects of life for adult females [2]. Additionally, studies using data from the National Ambulatory Medical Survey (NAMCS) database have identified substantial healthcare utilization for acne well beyond the teenage years [3].

Existing literature on acne among adult females and its treatment has focused on specific therapeutics, but investigation is needed of broader trends and treatments specific to adult female acne, which may have a pathogenesis and presentation different to juvenile acne [4, 5]. The pathogenesis of acne is not fully elucidated, though it involves sebocytes/sebum, follicular hyperkeratosis, bacteria, and inflammation [5, 6]. Age is of importance given the fluctuations and changes in hormonal balance in females from the third and fourth decades of life followed by significant estrogen drop-off with menopause. Therefore, our work aims to investigate national healthcare utilization patterns for acne management, comparing female and male population differences, and prescriptions within different age groups, specifically related to the adult female population.

## Methods

Using the 2002–2016 National Ambulatory Medical Care Survey (NAMCS) data, we studied patterns in office visits and treatment for females ages 13 years and older with acne in the United States by age category. Age categories defined began from adolescence, including ages 13–19 followed by stratification by decades. The database years were chosen based on full database availability for medication codes and provider specialty. Dermatology visits for acne were identified using ICD-9 codes 706.1 and ICD-10 codes L70.0 and L70.9. Sex of patients was based on NAMCS reporting, which included male or female. Additional demographic factors such as race, ethnicity, insurance types, and region were also based on NAMCS reporting.

We additionally analyzed the prescription of topical and systemic agents for females seeking care for acne by all providers, which included physicians and advanced practice providers in all specialties (including non-dermatology specialties such as primary care, family medicine, obstetrics and gynecology, etc.) for patients who had a primary, secondary, or tertiary diagnosis of acne. To characterize prescriptions, we identified medications based on acne treatment guidelines from American Academy of Dermatology. These included medication groups: systemic tetracycline antibiotics (eg. doxycycline and minocycline), spironolactone, combination oral contraceptive pills, topical tretinoin (tretinoin, adapalene, and tazarotene) only, topical antibiotic (eg. clindamycin or erythromycin) only, or combination topical tretinoin and antibiotic. Medications from the same drug class were combined and counted once for the encounter. Statistical analyses were performed using Stata 16 (StataCorp LLC). Descriptive statistics are reported as percentages, and χ^2^ was used for categorical comparisons. Graphs were created using Prism 9 (GraphPad Software).

## Results

There was a weighted total of 282,877,917 visits to dermatology for females above age 13 in the study period (**Table 1**). While 62.4–69.7% of office visits to dermatology were for acne regardless of sex during the teenage years, after age 20, there were significantly higher proportion of dermatology visits for acne among females compared to males (10.1% vs. 4.1%, P< 0.001). Among all dermatology office visits, a diagnosis of acne was made at 44.4% of visits for females ages 20–29, 22.9% for females ages 30–39, 12.4% for females ages 40–49, and decreased substantially at visits for females beyond age 50 (1.8%).

**Table 1:** Dermatology visits related to acne for female versus male patients.

Age groups	Total Dermatology Visits by Females	Diagnosis of Acne in Females	%	Total Dermatology Visits by Males	Diagnosis of Acne in Males	%	P value*
13–19	22,313,895	13,918,161	62.37	19,334,773	13,473,081	69.68	
20–29	24,561,556	10,899,975	44.38	13,942,660	3,637,344	26.09	<0.001
30–39	33,215,926	7,607,845	22.90	16,471,046	1,977,692	12.01	<0.001
40–49	39,504,477	4,890,284	12.38	22,461,687	1,128,227	5.02	<0.001
>50	163,191,246	2,875,150	1.76	145,500,032	1,306,024	0.90	<0.001
Total	282,877,917	40,191,415	14.21	217,710,198	21,522,368	9.88	<0.001

* Prevalence of dermatology visits related to acne were compared between males and females using χ2 tests.

Most of the female patients ages 13 and over with a diagnosis of acne were seen in a dermatology practice (70.6% of all visits) **(Table 2).** Patients seeking care in dermatology were predominantly White (85.2%), non-Hispanic (89.2%), and had private insurance (50.9%). Compared to other specialties, there was a greater proportion of patients with private insurance seeking dermatologic care for acne compared to other types of insurance. The overall proportion of adolescents seen for acne by specialties other than dermatology was greater (51.2%) than those seen (34.6%) by a dermatologist.

**Table 2:** Demographic information of female patients ages >13 seen for acne between 2002 to 2016 by specialty.

	All specialties		Dermatology			All other specialties*		
	Patient visits	%	Patient visits	%	% of all visits	Patient visits	%	% of all visits
**Total**	56,915,991	100.0	40,191,415	100.0	70.6	16,724,576	100.0	29.4
**Age groups**								
13–19	22,483,573	39.5	13,918,161	34.6		8,565,412	51.2	
20–29	14,213,438	25.0	10,899,975	27.1		3,313,463	19.8	
30–39	10,332,952	18.2	7,607,845	18.9		2,725,107	16.3	
40–49	6,196,478	10.9	4,890,284	12.2		1,306,193	7.8	
> 50	3,689,550	6.5	2,875,150	7.2		814,401	4.9	
**Patient Race**								
White	47,817,144	84.0	34,224,306	85.2		13,592,838	81.3	
Black	6,079,590	10.7	4,036,710	10.0		2,042,880	12.2	
Asian	2,831,050	5.0	1,800,203	4.5		1,030,846	6.2	
Other	188,207	0.3	130,196	0.3		58,011	0.3	
**Ethnicity**								
Hispanic	6,141,917	10.8	3,637,150	9.0		2,504,767	15.0	
Not Hispanic	49,924,347	87.7	35,832,362	89.2		14,091,985	84.3	
Unknown**	849,727	1.5	721,903	1.8		127,824	0.8	
**Insurance**								
Private	28,315,247	49.7	20,477,266	50.9		7,837,982	46.9	
Medicare	779,216	1.4	644,414	1.6		134,802	0.8	
Medicaid	2,929,379	5.1	1,158,099	2.9		1,771,280	10.6	
Self-pay	1,437,189	2.5	1,064,229	2.6		372,960	2.2	
Other	647,267	1.1	458,542	1.1		188,725	1.1	
Unknown**	22,807,693	40.1	16,388,865	40.8		6,418,828	38.4	

*All providers (MD, APRN, and PAs) and specialties other than dermatology (family medicine, pediatrics, primary care, general surgery, and more) were included in the analysis.

**Data unavailable in the dataset.

**Fig 1:**
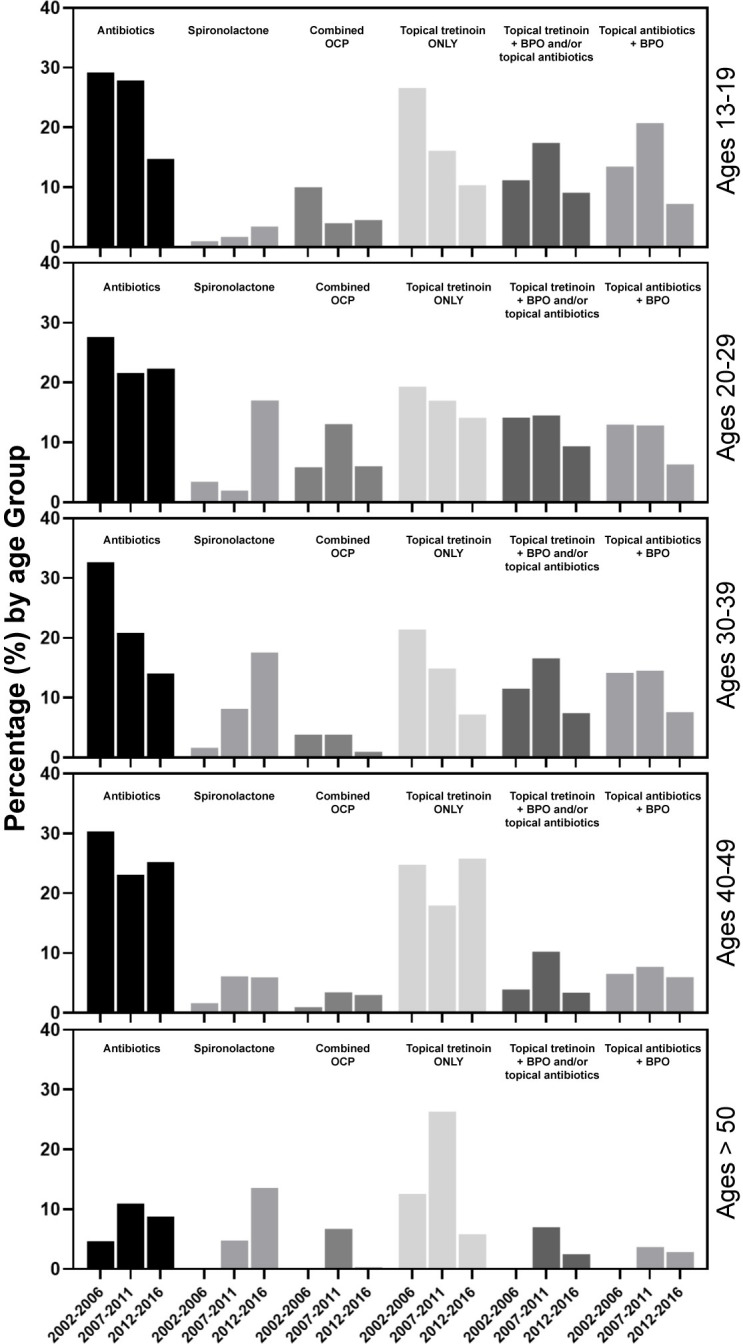
Prescription trends for each age group by drug class and years. Each plot is proportion of female patients in the age group prescribed the medication class out of the total number of patients in the age group and years.

Overall, tetracycline-class antibiotics were the most prescribed therapy within all age groups of females with acne by all providers (22.9% of visits by all females) **(Table 3).** Compared to earlier time periods, however, there was a substantial rise in prescriptions of spironolactone from 2012–2016 for females ages 20–29 and 30–39 (**Fig 1)**. Between 2012–2016, spironolactone rose to become the second most prescribed treatment for females ages 20–29 and the most prescribed treatment for females ages 30–39 and >50. Females ages 20–29 received combination birth control at higher rates (8.8%) than any other age group. Compared to females ages 30–39, females ages 40–49 received spironolactone (9.0% vs. 4.7%) and topical antibiotics (12.1% vs. 6.8%) at lower rates and received oral antibiotics at a higher rate (22.6% vs. 26.0%).

**Table 3:** Treatments prescribed for adult female acne by age group for all visits related to acne between 2002 to 2016*.

	All patients	Antibiotics (minocycline, tetracycline, doxycycline)	Spironolactone	Combination birth control (estrogen + progesterone)	Topical tretinoin ONLY	Topical tretinoin + BPO/ Topical Abx	Topical Antibiotic (clindamycin or erythromycin) + BPO
Age group	Females with Diagnosis of Acne						
13–19	22,483,573 (100)**	5,366,545 (23.9)	458,741 (2.0)	1,410,550 (6.3)	4,005,921 (17.8)	2,787,079 (12.4)	3,050,658 (13.6)
2002–2006	7,846,817	2,292,054 (29.2)	77,758 (1.0)	785,975 (10.0)	2,089,779 (26.6)	876,669 (11.2)	1,054,846 (13.4)
2007–2011	6,960,349	1,941,138 (27.9)	119,266 (1.7)	278,380 (4.0)	1,121,514 (16.1)	1,212,363 (17.4)	1,442,414 (20.7)
2012–2016	7,676,406	1,133,352 (14.8)	261,718 (3.4)	346,195 (4.5)	794,628 (10.4)	698,048 (9.1)	553,398 (7.2)
20–29	14,213,438 (100)	3,362,905 (23.7)	977,580 (6.9)	1,246,339 (8.8)	2,392,481 (16.8)	1,828,371 (12.9)	1,556,314 (10.9)
2002–2006	4,315,534	1,193,030 (27.6)	147,069 (3.4)	252,075 (5.8)	833,182 (19.3)	610,220 (14.1)	560,230 (13.0)
2007–2011	5,674,298	1,226,648 (21.6)	111,131 (2.0)	739,592 (13.0)	963,293 (17.0)	823,246 (14.5)	729,093 (12.8)
2012–2016	4,223,606	943,228 (22.3)	719,381 (17.0)	254,672 (6.0)	596,006 (14.1)	394,906 (9.3)	266,991 (6.3)
30–39	10,332,952 (100)	2,337,454 (22.6)	933,473 (9.0)	297,434 (2.9)	1,505,014 (14.6)	1,225,345 (11.9)	1,253,196 (12.1)
2002–2006	3,487,813	1,139,731 (32.7)	56,608 (1.62)	132,638 (3.8)	746,817 (21.4)	401,442 (11.5)	493,613 (14.2)
2007–2011	3,458,486	721,399 (20.9)	281,944 (8.2)	132,190 (3.8)	514,445 (14.9)	572,996 (16.6)	502,358 (14.5)
2012–2016	3,386,653	476,324 (14.1)	594,920 (17.6)	32,607 (1.0)	243,752 (7.2)	250,907 (7.4)	257,225 (7.6)
40–49	6,196,478 (100)	1,614,132 (26.0)	290,075 (4.7)	158,247 (2.6)	1,397,384 (22.6)	379,267 (6.1)	421,829 (6.8)
2002–2006	1,915,359	582,084 (30.4)	31,517 (1.6)	18,599 (1.0)	474,707 (24.8)	74,963 (3.9)	125,439 (6.5)
2007–2011	2,318,104	536,367 (23.1)	141,793 (6.1)	80,243 (3.5)	416,030 (17.9)	237,949 (10.3)	179,007 (7.7)
2012–2016	1,963,014	495,682 (25.3)	116,765 (5.9)	59,405 (3.0)	506,646 (25.8)	66,355 (3.4)	117,383 (6.0)
> 50	3,689,550 (100)	326,172 (8.8)	270,421 (7.3)	104,295 (2.8)	570,703 (15.5)	140,852 (3.8)	96,793 (2.6)
2002–2006	737,001	34,425 (4.7)	0 (0)	0 (0)	92,660 (12.6)	0 (0)	0 (0)
2007–2011	1,491,626	163,106 (10.9)	71,958 (4.8)	100,080 (6.7)	392,368 (26.3)	104,750 (7.0)	54,987 (3.7)
2012–2016	1,460,923	128,640 (8.8)	198,463 (13.6)	4,215 (0.3)	85,676 (5.9)	36,102 (2.5)	41,806 (2.9)
Total	56,915,991 (100)	13,007,208 (22.9)	2,930,291 (5.1)	3,216,866 (5.7)	9,871,503 (17.3)	6,360,914 (11.2)	6,378,789 (11.2)

*All providers (MD, APRN, and PAs) and specialties (family medicine, pediatrics, primary care, general surgery, and more) were included in the analysis of patterns.

** Data are presented as number (%). The highlighted totals are based on the patient population in the age group while the total on the last line are based on all patients.

## Discussion

Our analysis expands on previous research by analyzing the differences in healthcare utilization for acne by sex and age and temporal patterns in prescribed treatments for acne among females by age group. While previous studies have investigated the self-reported prevalence of acne by age groups, this is the first study to analyze healthcare utilization and treatment for acne stratified by age and sex [1]. Specifically, our analysis demonstrates that there is a substantial number of dermatology visits by adult females aged 20 years and older, including those females aged 50 years and older, for acne. In addition, in our study, there was a statistically significant difference in the proportion of dermatology visits that were for acne among adult females versus adult males. This finding is consistent with previous studies showing that acne is less common in adults males when compared to adult females [7, 8]. However, it was surprising to see that acne accounted for more than a 1.5-fold higher proportion of dermatology visits among females compared to males ages 20–29 given that prior studies show more subtle differences in acne prevalence between these sexes in that age range [1]. We hypothesize that sex differences in dermatologic care utilization for acne may be tied to increased severity of acne among adult females as well as increased impact on quality of life in this population [7, 8]. Our study additionally complements a past study that suggested the rate of adult acne has not increased but that the average age of patients seeking care has been rising [3].

Consistent with prior work, broad-spectrum tetracycline-class antibiotics were the most prescribed treatment for females with acne during our overall study period. However, we also demonstrate evidence that the antiandrogenic agent spironolactone is increasingly used for the treatment of acne among adult females. While a previous study showed that spironolactone prescriptions were on the rise between 2004–2013, we found that spironolactone became the predominant form of treatment for acne among certain age groups of females in the 2012–2016 time period [7]. Females ages 30–39 were prescribed spironolactone more frequently than systemic antibiotics in the years 2012–2016, which may demonstrate an increasing trend towards use of antiandrogenic agents for acne in this age group.

While pathogenesis of any type of acne is multifactorial, it is postulated that adult and adolescent acne may involve divergent etiologies. Adult acne is often associated with genetics, exacerbation around menstrual cycles, and stress [8–11]. The leading hypothesis based on *in-vivo* studies on sebaceous glands and clinical data have suggested that increased androgen production results in excessive and altered production of sebum, leading to cutaneous dysbiosis [12–16]. However the hormonal screening of women with adult acne usually reveals normal levels of DHEA, testosterone, and androgen, demonstrating a potential intracrine effect of androgen [17, 18]. Though much of the *in vivo* work has focused on localizing androgen receptors in human skin, some preclinical studies demonstrated that a significant amount of estrogen can reduce the size of sebaceous glands and inhibit sebum production [16, 19]. In addition, the pathogenesis of acne in some females may be related to the use of progesterone-only oral contraceptives, which have been reported to exacerbate acne–these findings suggest progesterone may act on the pilosebaceous unit in unknown ways [22]. Further studies on the pathogenesis of adult female acne may lead to better treatments for this patient population.

While our study shows that tetracycline antibiotics remain commonly used for adult female acne, they are associated with high antibiotic failure rates, with previously reported failure rates up to 81% [17, 20]. Frequent and prevalent use of broad-spectrum tetracycline antibiotics can lead to resistance and treatment failure. Antibiotic stewardship may improve over time as the benefits of antiandrogenic agents in female adult acne and narrow-spectrum antibiotics, such as sarecycline, in reducing gut microbiome dysbiosis and antibiotic resistance are understood [21].

There are limitations to this study. First, recent acne treatment guidelines from the American Academy of Dermatology were released in 2016 [22]. Our study period included 2002–2016 due to the availability of NAMCS dataset; thus, our study period may not have captured changing practice patterns in response to the guidelines. Additionally, differences in the use of antibiotic acne medications and antihormonal medications may differ based on specialty/practice provider type [23]. It is also possible that the systemic agents analyzed may have been prescribed for other indications such as polycystic ovary syndrome, dysmenorrhea, or contraception in the case of combination birth control pills. Lastly, we were not able to reliably study isotretinoin due to its inconsistent representation in the dataset.

Given our data showing the substantial proportion of adult females who seek dermatologic care for acne in comparison to adult males and changing treatment trends for adult female acne in recent years, there is a clear need for further studies investigating the pathogenesis of and comparative effectiveness of therapies specifically for adult female acne.
